# Bypass of Abasic Site–Peptide Cross-Links by Human Repair and Translesion DNA Polymerases

**DOI:** 10.3390/ijms241310877

**Published:** 2023-06-29

**Authors:** Anna V. Yudkina, Alexander E. Barmatov, Nikita A. Bulgakov, Elizaveta O. Boldinova, Evgeniy S. Shilkin, Alena V. Makarova, Dmitry O. Zharkov

**Affiliations:** 1 SB RAS Institute of Chemical Biology and Fundamental Medicine, Novosibirsk 630090, Russia; barmatov@niboch.nsc.ru (A.E.B.); bulgakov@niboch.nsc.ru (N.A.B.); 2 Institute of Molecular Genetics, National Research Center “Kurchatov Institute”, Moscow 123182, Russia; lizaboldinova@yandex.ru (E.O.B.); shilkinevgeniy.chem@gmail.com (E.S.S.); amakarova-img@yandex.ru (A.V.M.); 3 Department of Natural Sciences, Novosibirsk State University, Novosibirsk 630090, Russia

**Keywords:** DNA damage, DNA repair, DNA–peptide cross-links, AP sites, DNA polymerases, translesion synthesis, DNA lesion bypass, mutagenesis

## Abstract

DNA–protein cross-links remain the least-studied type of DNA damage. Recently, their repair was shown to involve proteolysis; however, the fate of the peptide remnant attached to DNA is unclear. Particularly, peptide cross-links could interfere with DNA polymerases. Apurinuic/apyrimidinic (AP) sites, abundant and spontaneously arising DNA lesions, readily form cross-links with proteins. Their degradation products (AP site–peptide cross-links, APPXLs) are non-instructive and should be even more problematic for polymerases. Here, we address the ability of human DNA polymerases involved in DNA repair and translesion synthesis (POLβ, POLλ, POLη, POLκ and PrimPOL) to carry out synthesis on templates containing AP sites cross-linked to the N-terminus of a 10-mer peptide (APPXL-I) or to an internal lysine of a 23-mer peptide (APPXL-Y). Generally, APPXLs strongly blocked processive DNA synthesis. The blocking properties of APPXL-I were comparable with those of an AP site, while APPXL-Y constituted a much stronger obstruction. POLη and POLκ demonstrated the highest bypass ability. DNA polymerases mostly used dNTP-stabilized template misalignment to incorporate nucleotides when encountering an APPXL. We conclude that APPXLs are likely highly cytotoxic and mutagenic intermediates of AP site–protein cross-link repair and must be quickly eliminated before replication.

## 1. Introduction

Apurinic/apyrimidinic sites (AP sites) are frequently encountered spontaneous DNA lesions that occur in the human genome at the steady-state level of ~20,000 per cell under normal conditions, with their number increasing significantly when cells are exposed to genotoxic stress [1,2]. Recent studies have shown that “AP sites” are not limited to their best-known representative, the aldehydic AP site, but are actually a group of structurally related abasic lesions that may be oxidized at the C1′, C2′, C4′ or C5′ positions of the deoxyribose ring [3,4,5,6,7]. AP sites and their derivatives are highly electrophilic and readily react with nucleophilic groups including amines and thiols in proteins, thus forming a variety of DNA–protein cross-links (DPXLs). It has been reported that AP sites covalently trap DNA repair proteins such as DNA polymerases β (POLβ) and λ (POLλ) [8,9], DNA glycosylases such as endonuclease III [10,11], formamidopyrimidine–DNA glycosylase, endonuclease VIII-like protein 1 [11], *O*^6^-alkylguanine-DNA alkyltransferase [12] and DNA ligase [13], as well as abundant intracellular proteins including histones [14,15,16], chaperones [17] and ribosomal proteins [17,18]. Recent developments in mass-spectrometry-based methods have produced estimates of the background level of AP site–protein cross-links at ~40 per 10^6^ nucleotides [19,20], which is comparable with the levels of unadducted AP sites and major oxidative DNA lesions such as 8-oxoguanine [2,21].

Investigations into the mechanisms of repair of DPXLs have gained fresh momentum in the last few years, after it was discovered that such adducts are cleaved by proteases to small peptide remnants attached to DNA [22,23,24,25]. Nevertheless, the effects of these residual fragments of DPXLs on normal replication, as well as the mechanism of their repair after proteolysis, are still not clear [26]. It is assumed that SPRTN1, the main protease involved in the replication-coupled degradation of DPXLs, processes the cross-link to peptides which are short enough to be bypassed by translesion DNA polymerases [25], since large peptide adducts will presumably block DNA synthesis. In vitro experiments have shown that small peptides (from tetra- to dodecapeptide) are quite efficiently bypassed by translesion DNA polymerases [27,28,29,30], while large peptides (for example, 23-mer peptides) block synthesis even by translesion DNA polymerases [29]. These observations disagree with another report that even a large 31-mer peptide can be bypassed by translesion DNA polymerases κ and η [31]. Despite the somewhat inconsistent data in the literature, it is generally accepted that the bypass of a DNA–peptide adduct is mainly dependent on its size. Presumably, small peptides can fit neatly into the large groove of DNA, thereby facilitating their passage by polymerases. In vivo studies have demonstrated that the mutagenic bypass of peptide adducts requires translesion DNA polymerases [31,32].

The mechanism of coordination between the proteolysis of the cross-linked protein and translesion DNA synthesis has not been elucidated so far. If large peptides can be bypassed by translesion DNA polymerases, albeit with a lower rate and efficiency [25], blocks caused by DNA–peptide adducts can potentially be resolved before complete proteolysis of the cross-linked protein. On the other hand, a translesion DNA polymerase stalled at the replication fork can induce undesirable events such as template switching with the formation of chicken foot structures that require complex recombination repair to resume replication [33,34].

As the repair of AP site–protein cross-links in human cells proceeds through proteolysis, it can be assumed that most of these adducts would at some point be converted to AP site–peptide adducts, and therefore significant amounts of the latter would be present in the cell, ready for an encounter with DNA polymerases. However, despite the prevalence of cross-links conjugated through AP sites [19,20], this type of lesion has not yet been studied. All models of DNA–peptide cross-links were obtained via the conjugation of peptides with DNA bases, either normal or pre-derivatized with reactive moieties. The studied systems modeling natural cross-links include conjugates of peptides through the exocyclic amino groups of guanine and adenine modified with acrolein [28,35] or 1,2-dibromoethane [36], conjugates through the carbonyl moiety of 5-formylcytosine [31,32] and conjugates with γ-hydroxypropanoguanine [27,37]. In addition, synthetic adducts were used as model systems, such as products of click chemistry [30] and reduced Schiff base adducts to 7-deaza-7-(2-oxoethyl)guanine [38]. Almost all studies of DNA–peptide conjugates have been carried out with N-terminally adducted linear peptides, while the proteolysis of DPXLs is expected to form conjugates at the internal positions of peptides.

**Figure 1:**
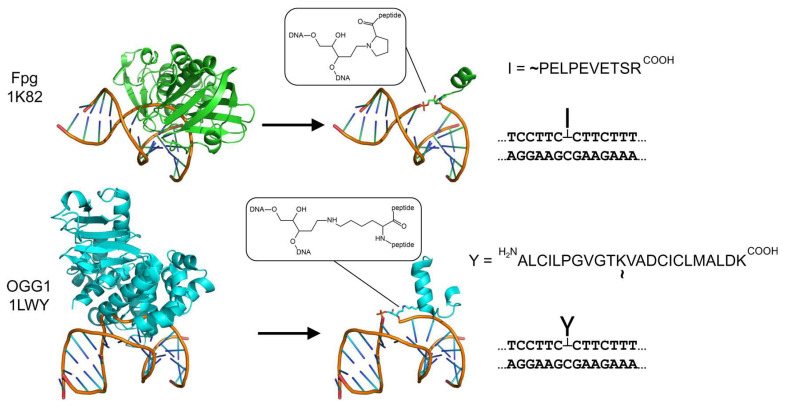
Scheme of APPXL substrates. *E. coli* Fpg and human OGG1 are cross-linked by NaBH_4_ to a DNA duplex containing a single 8-oxoguanine residue; as a result, a stable AP site–protein cross-link is formed. After extensive trypsinolysis, Fpg yields an N-terminally cross-linked 10-mer peptide (APPXL-I) and OGG1, an internally cross-linked 23-mer peptide (APPXL-Y). Structures shown are those of the NaBH_4_-trapped AP site–protein cross-links with *E. coli* Fpg [40] and human OGG1 [41]. In the peptide sequences, a tilde marks the cross-linking site.

Recently, we developed a method to obtain model AP site–peptide cross-links (APPXLs) of different structures based on the trypsinolysis of trapped covalent intermediates of DNA glycosylases Fpg and OGG1 with DNA [39] (Figure 1). In the resulting adducts, an AP site is stably coupled with either a 10-mer peptide through its N-terminal amino group (APPXL-I adduct) or a 23-mer peptide through an ε-amino group of internal Lys residue (APPXL-Y adduct; names -I and -Y are due to the visual resemblance to the terminally and internally attached peptide, respectively; Figure 1). The adducts can either be incorporated into the DNA template or, if necessary, into a downstream strand displaced by DNA polymerase during synthesis. These APPXLs turned out to completely block African swine fever virus DNA polymerase PolX and strongly block the Klenow fragment of *E. coli* DNA polymerase I and bacteriophage DNA polymerase RB69, but they were partly bypassed by translesion DNA polymerase IV from *Sulfolobus solfataricus*. We have also tested the miscoding properties of these DNA polymerases and demonstrated that DNA polymerases KF and RB69 follow the “A-rule” during the synthesis opposite the non-coding APPXLs, while PolX and Dpo4 use the template misalignment mechanism to select the incorporated dNMP. Therefore, in the model systems, APPXLs show the properties of cytotoxic (blocking) and mutagenic (miscoding) lesions. To extend our understanding of possible mechanisms of APPXL bypass and APPXL-induced mutagenesis in the human system, here, we use the same model to investigate the miscoding and blocking properties of these adducts for several human translesion DNA polymerases, namely DNA polymerase η (POLη), κ (POLκ) and primase–polymerase (PrimPOL), and major DNA repair polymerases β (POLβ) and λ (POLλ).

## 2. Results

### 2.1. Repair DNA Polymerases POLβ and POLλ Are Blocked by AP Site–Peptide Cross-Links

Family X DNA polymerases are small monomeric enzymes with a primary function of filling short gaps arising during DNA repair. Structurally and catalytically, these DNA polymerases share relatively low processivity, high affinity for gapped DNA facilitated by their accessory 2′-deoxyribo-5′-phosphate lyase domain and a lack of 3′→5′ exonuclease activity. The best-characterized members of Family X are POLβ and POLλ, which participate in the base excision repair (BER) pathway. POLβ is the main polymerase involved in the BER, while POLλ likely plays a backup role. BER polymerases usually encounter lesions while repairing clustered DNA damage containing modified nucleotides in the opposite DNA strands [42,43]. Here, we addressed the interaction of POLβ and POLλ with APPXLs.

**Figure 2:**
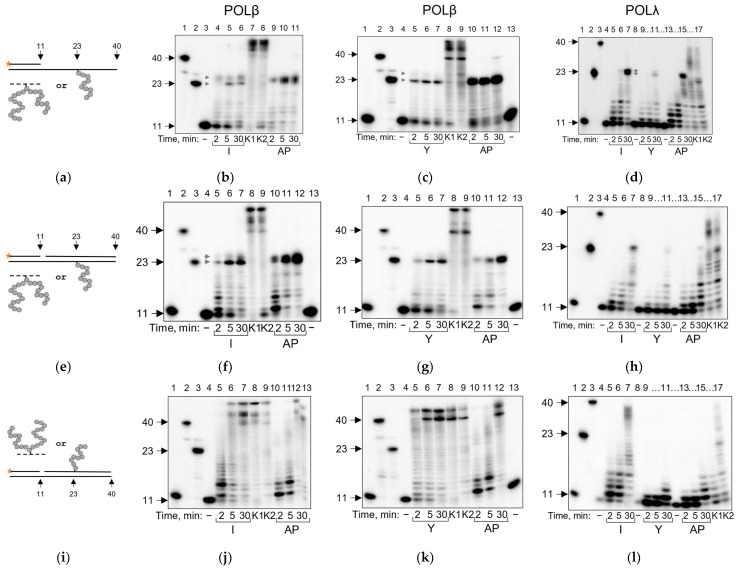
Running start synthesis by POLβ and POLλ on APPXL-containing substrates. Primer–template substrate, and adduct in the template (**a**); representative gels showing extension for POLβ and APPXL-I (**b**); POLβ and APPXL-Y (**c**); POLλ and both APPXLs (**d**). Primer–template–downstream strand substrate, and adduct in the template (**e**); representative gels showing extension for POLβ and APPXL-I (**f**); POLβ and APPXL-Y (**g**) and POLλ and both APPXLs (**h**). Primer–template–downstream strand substrate, and adduct in the downstream strand (**i**); representative gels showing extension for POLβ and APPXL-I (**j**); POLβ and APPXL-Y (**k**) and POLλ and both APPXLs (**l**). The nature of the substrate and reaction time are indicated below the gel image; I, APPXL-I; Y, APPXL-Y; AP, natural AP site. K1, undamaged primer–template substrate; K2, undamaged primer–downstream strand–template substrate; “−”, no enzyme added. In all gels: lanes 1–3, size markers with lengths corresponding to the primer (11 nt), full-size product (40 nt) and primer extended to the site of cross-linking (23 nt). Gray arrows indicate synthesis pause points.

First, we tested the ability of DNA polymerases to bypass the AP–peptide obstacle in the running start conditions while the 3′-OH primer terminus was located upstream at a distance (12 bases) from the site of the cross-link (Figure 2a). This should allow the polymerase to bind the substrate and begin synthesis with little interference from the peptide part of the cross-link. As expected, both POLβ and POLλ were blocked by APPXL-I located in the template strand of single-stranded DNA (Figure 2b–d). We observed an accumulation of the 23 and 24 nucleotides long products, reflecting inefficient nucleotide incorporation opposite the cross-link site (23-nt) and inefficient extension of any product of incorporation (24-nt). This pattern is quite typical during DNA synthesis on the templates containing non-coding damage [44,45,46]. The branched APPXL-Y adduct demonstrated the same blocking properties; however, with this peptide, we observed reduced primer utilization for both DNA polymerases (Figure 2c,d). Possibly, the larger size of the Y-peptide (23 amino acid residues) distorts the DNA structure and complicates primer binding by DNA polymerases even in the running start construct.

POLβ and POLλ prefer gapped substrates to simple primer–template substrates to insert a single nucleotide; however, their ability to displace the downstream strand is limited [47,48,49,50]. The presence of the downstream strand (Figure 2e) reduced the ability of POLβ and POLλ to bypass the APPXL obstacles (Figure 2f–h). Interestingly, however, when APPXLs were located in the downstream strand rather than in the template (Figure 2i), POLβ displaced the strand carrying either adduct better than the AP-site-containing strand (Figure 2j,k), while POLλ showed facilitated strand displacement only for APPXL-I (Figure 2l). This may indicate that APPXLs destabilize the DNA duplex.

### 2.2. POLβ and POLλ Use dNTP-Stabilized Misalignment to Incorporate Nucleotides Opposite AP Site–Peptide Cross-Links

**Figure 3:**
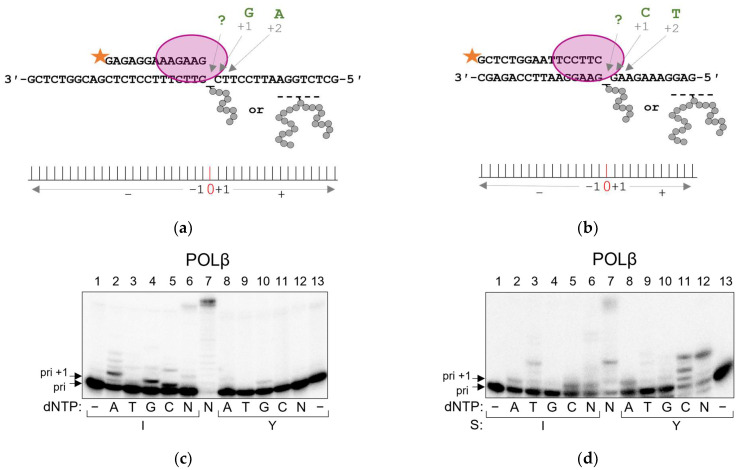

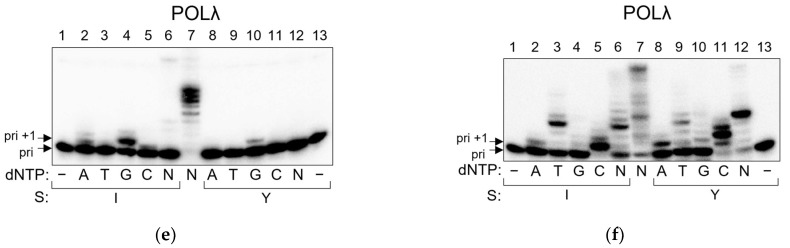
Incorporation preference of POLβ and POLλ in standing start synthesis on APPXL-containing substrates. Schemes of the substrates are shown above the gel images. Pyrimidine-rich template (**a**); representative gels for nucleotide incorporation by POLβ (**c**) and POLλ (**e**). Purine-rich template (**b**); representative gels for nucleotide incorporation by POLβ (**d**) and POLλ (**f**). The nature of the substrate and dNTP are indicated below the gel image; I, APPXL-I; Y, APPXL-Y; N, mixture of all four dNTPs. K1, undamaged primer–template substrate; K2, undamaged primer–downstream strand–template substrate. In all gels: lane 7, undamaged substrate; “−”, no enzyme added.

To investigate the miscoding potential of APPXLs for POLβ and POLλ, we switched to the standing start DNA polymerase assay, where the 3′-end of the primer is located immediately before the damaged site and a single dNTP is present in the reaction. DNA polymerases bind the primer much less efficiently in the vicinity of the bulky peptide obstacle and thus have to be used in higher concentrations. Nevertheless, the standing start scheme offers a way to estimate the mutagenic potential of the damage, revealing the DNA polymerases’ dNMP incorporation preference. On the model substrate with the pyrimidine-rich template sequence identical to that used in the running start experiments (Figure 3a), POLβ demonstrated a preference for nucleotide incorporation opposite APPXLs in the order G > A > C > T for both I- and Y-peptides (Figure 3c). The overall efficiency of the nucleotide incorporation opposite APPXL-Y was lower than opposite APPXL-I, which was likely due to poorer DNA binding. POLλ behaved similarly to POLβ, showing the preference of nucleotide incorporation opposite APPXLs in the order G >> A > C > T (Figure 3e).

Family X DNA polymerases are known for their strong propensity for “skipping” the AP site lesion using the dNTP-stabilized misalignment mechanism, in which the polymerase bends DNA and incorporates the nucleotide complementary to the next position in the template [51,52,53]. Thus, to distinguish between the intrinsic polymerases’ preference for dGMP incorporation opposite APPXLs and the misalignment mechanism, we designed the second substrate of a different sequence (purine-rich template) for the standing start assay (Figure 3b). Provided with this model substrate, both POLβ and POLλ changed their preferences of incorporation. POLβ’s preference of dNMP incorporation opposite APPXLs became C > A > T > G (Figure 3d), leading us to propose that POLβ uses the +1 dNTP-stabilized misalignment mechanism to overcome APPXLs and, to a lesser extent, incorporates dAMP opposite APPXL. POLλ changed its preference for nucleotide incorporation even more dramatically to C >> T > A > G (Figure 2f), indicating a strong tendency to skip APPXLs by +1 and +2 dNTP-stabilized misalignment rather than by incorporating a nucleotide opposite the APPXL. Interestingly, POLλ skipped a bulkier APPXL-Y even easier than the APPXL-I adduct.

### 2.3. POLκ Can Bypass AP Site–Peptide Cross-Links

In the living cell, copying damaged DNA, or translesion DNA synthesis, involves a plethora of specialized DNA polymerases. Most of them belong to the structural Family Y, whose members possess a wide and relaxed active site capable of accommodating non-canonical nucleotides in the template. In a generally accepted two-polymerase model of translesion synthesis, Y-family DNA polymerases (“inserters”) incorporate some dNMP opposite a damaged base, and then another DNA polymerase (“extender”, often DNA polymerase ζ belonging to Family B) adds nucleotides to the new primer terminus past the lesion. Here, we studied the interaction of human Family Y DNA polymerases with APPXLs.

**Figure 4:**
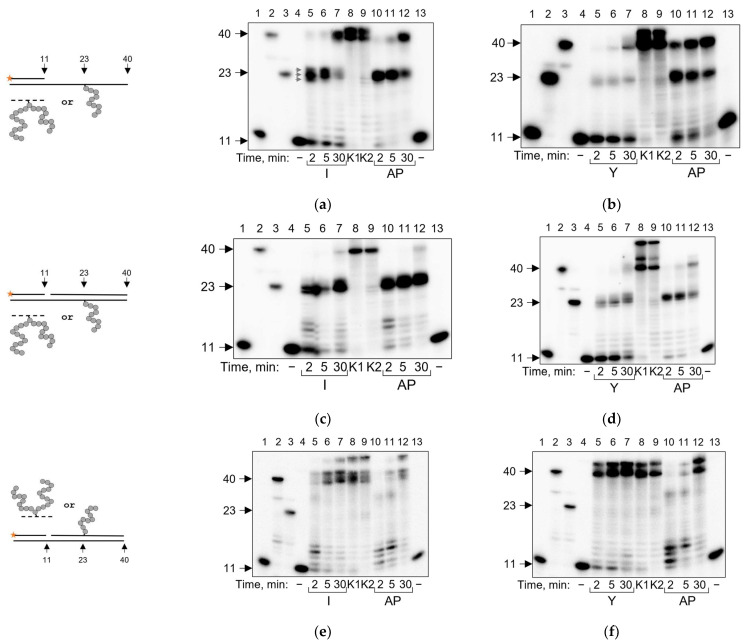
Running start synthesis by POLκ on APPXL-containing substrates. Schemes of the substrates are shown to the left of the gel images. Primer–template substrate, and adduct in the template: (**a**) APPXL-I; (**b**) APPXL-Y. Primer–template–downstream strand substrate, and adduct in the template: (**c**) APPXL-I; (**d**) APPXL-Y. Primer–template–downstream strand substrate, and adduct in the downstream strand: (**e**) APPXL-I; (**f**) APPXL-Y. The nature of the substrate and reaction time are indicated below the gel image; I, APPXL-I; Y, APPXL-Y; AP, natural AP site. K1, undamaged primer–template substrate; K2, undamaged primer–downstream strand–template substrate; “−”, no enzyme added. In all gels: lanes 1–3, size markers with lengths corresponding to the primer (11 nt), full-size product (40 nt) and primer extended to the site of cross-linking (23 nt). Gray arrows indicate synthesis pause points.

POLκ is a translesion DNA polymerase specialized in overcoming adducts exposed to the minor groove of DNA and is often inhibited by major groove adducts [54,55,56]. POLκ incorporates ~20–30 nucleotides per binding event [57]. Here, we observed POLκ effectively bypassing APPXL-I (~42% bypass in 30 min) at approximately the same extent as an AP site (~43–45% bypass in 30 min) (Figure 4a). Along with the accumulation of a full-sized product, we observed strong synthesis pause points at and before the damaged site. APPXL-Y was bypassed by POLκ less efficiently; however, as with POLβ and POLλ, we again observed reduced primer utilization in the reaction with APPXL-Y (Figure 4b). Thus, it is not clear whether the lower percent of APPXL-Y bypass is due to internal properties of the adduct in the active site of POLκ or to the limited ability of the enzyme to bind the substrate initially.

POLκ has limited ability for strand displacement [58]; accordingly, the damage bypass was somewhat suppressed in the presence of a downstream strand (~12–30% AP site bypass in 30 min). However, we observed a similar ability of POLκ to overcome APPLXs during the displacement synthesis: ~12% of bypass for APPXL-I (Figure 4c) and ~19% of bypass for APPXL-Y (Figure 4d). POLκ’s ability to displace a strand containing the adduct increased in the order AP site < APPXL-I < APPXL-Y (Figure 4e,f), recapitulating the results with Family X polymerases.

**Figure 5:**
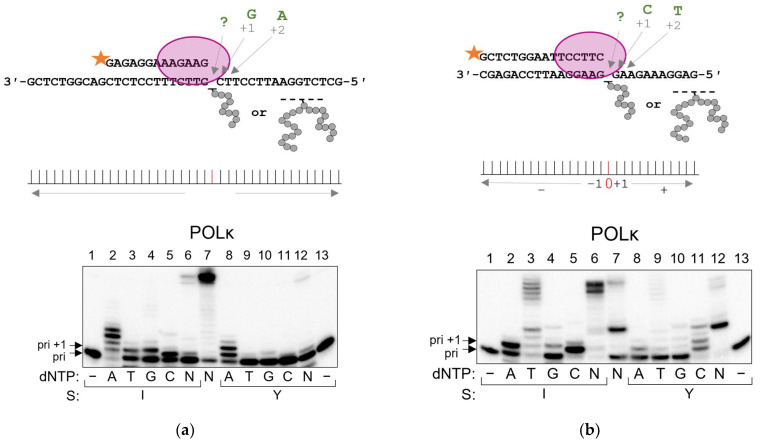
Incorporation preference of POLκ in standing start synthesis on APPXL-containing substrates. Schemes of the substrates are shown above the gel images. (**a**) Pyrimidine-rich template, (**b**) purine-rich template. The nature of the substrate and dNTP are indicated below the gel image; I, APPXL-I; Y, APPXL-Y; N, mixture of all four dNTPs. K1, undamaged primer–template substrate; K2, undamaged primer–downstream strand–template substrate. In all gels: lane 7, undamaged substrate; “−“, no enzyme added.

Under the standing start conditions, POLκ predominantly incorporated dAMP opposite APPXLs, and the general preference of nucleotide incorporation was in the order A > G,C > T (Figure 5a). However, this preference changed after switching to the alternative substrate: C > A,T > G (Figure 5b). This may suggest that, rather than strictly adhering to a single mechanism of peptide cross-link bypass, POLκ uses both the template misalignment mechanism (+1 more efficiently than +2) and the incorporation of dAMP opposite the lesion. The cumulative effect of dAMP incorporation opposite the lesion and +2 misalignment would explain the preference of POLκ for A on the pyrimidine-rich template, while dAMP incorporation alone opposite the lesion would show up as a secondary contribution of A on the purine-rich template. However, it should be kept in mind that the specific mechanisms of APPXL interactions with POLκ in the standing start mode can be influenced by the complicated primer end binding, and may be different during processive synthesis.

### 2.4. POLη Can Bypass AP Site–Peptide Cross-Links with Indiscriminate dNMP Incorporation

POLη is an archetypical eukaryotic Family Y translesion DNA polymerase that plays an important role in maintaining genome stability after UV-induced DNA damage [59,60]. Moreover, POLη is shown to bypass several bulky adducts, such as cisplatin G^G intrastrand cross-links [61], benz[*a*]pyrene [62,63], etc. Several studies have shown that POLη could be responsible for AP site bypass with G or A incorporation in vitro [61,64,65] and in cells [66]. Therefore, structural features of POLη allow it to incorporate a dNMP opposite both large and conformationally restrained lesions and opposite the non-coding AP site, which makes it a promising candidate for APPXL bypass.

**Figure 6:**
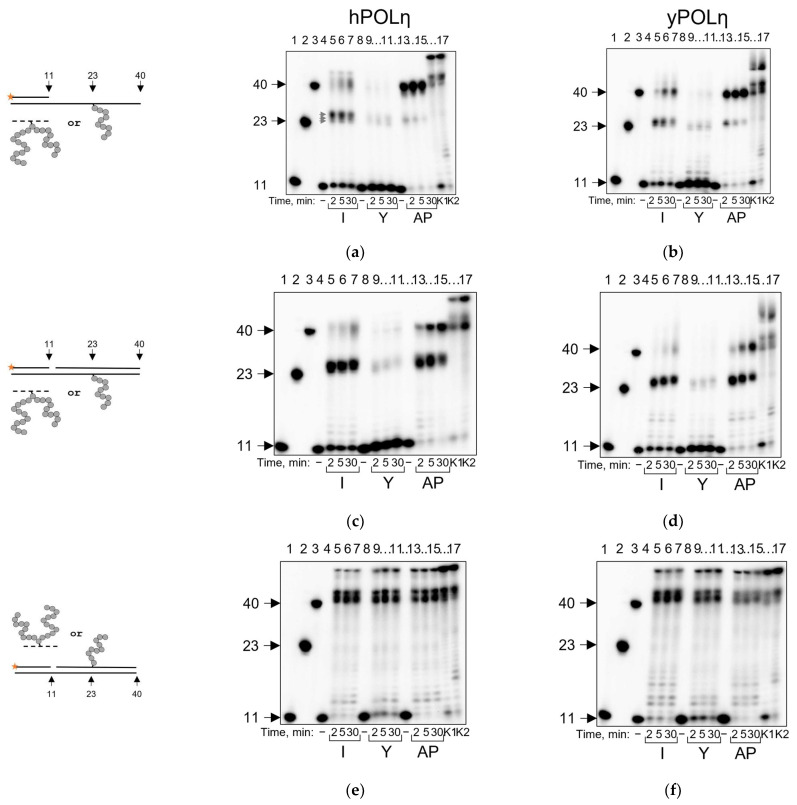
Running start synthesis by hPOLη and yPOLη on APPXL-containing substrates. Schemes of the substrates are shown to the left of the gel images. Primer–template substrate, and adduct in the template: (**a**) hPOLη; (**b**) yPOLη. Primer–template–downstream strand substrate, and adduct in the template: (**c**) hPOLη; (**d**) yPOLη. Primer–template–downstream strand substrate, and adduct in the downstream strand: (**e**) hPOLη; (**f**) yPOLη. The nature of the substrate and reaction time are indicated below the gel image; I, APPXL-I; Y, APPXL-Y; AP, natural AP site. K1, undamaged primer–template substrate; K2, undamaged primer–downstream strand–template substrate; “−”, no enzyme added. In all gels: lanes 1–3, size markers with lengths corresponding to the primer (11 nt), full-size product (40 nt) and primer extended to the site of cross-linking (23 nt). Gray arrows indicate synthesis pause points.

POLη, despite being used in lower concentrations than other polymerases in this study, easily bypassed AP sites and synthesized the full-size product (>95% bypass in 30 min) (Figure 6a). It was also able to bypass APPXL-I and APPXL-Y; however, the efficiency of the peptide adducts bypass was drastically reduced (Figure 6a). Along with a significant amount of the intact primer, 2–3 synthesis pause points were observed. Interestingly, they corresponded to 25-mer, 26-mer and 27-mer products of the synthesis, which means that POLη incorporates nucleotides opposite the adduct efficiently but has difficulty in extending past the lesion (Figure 6a). To confirm these results, we also tested yeast POLη under the running start conditions and observed the same ability for damage bypass and accumulation of the products of synthesis beyond the cross-link site (Figure 6b).

POLη is relatively processive and capable of strand displacement [67]. The presence of the displaced strand shifted the product distribution for both hPOLη and yPOLη from full-size to the accumulation of products stalled near the lesion, while maintaining the general termination pattern observed for the single-stranded substrate (Figure 6c,d). If the substrates contained APPXLs in the displaced strand, the accumulation of the full-size products proceeded without any noticeable pause points (Figure 6e,f).

**Figure 7:**
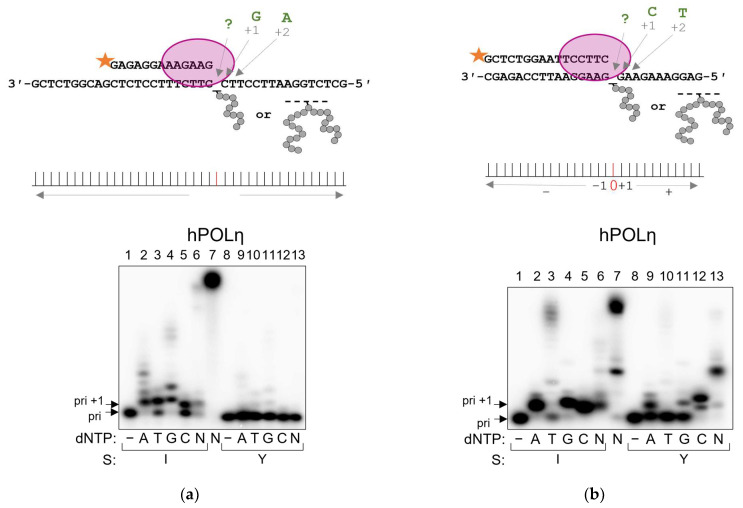

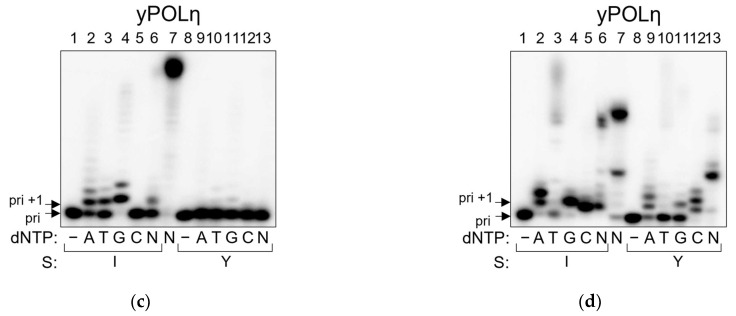
Incorporation preference of hPOLη and yPOLη in standing start synthesis on APPXL-containing substrates. Schemes of the substrates are shown above the gel images. Pyrimidine-rich template: (**a**) hPOLη; (**c**) yPOLη. Purine-rich template: (**b**) hPOLη; (**d**) yPOLη. The nature of the substrate and dNTP are indicated below the gel image; I, APPXL-I; Y, APPXL-Y; N, mixture of all four dNTPs. K1, undamaged primer–template substrate; K2, undamaged primer–downstream strand–template substrate. In all gels: lane 7, undamaged substrate; “−“, no enzyme added.

Standing start experiments revealed the superior ability of POLη to process 3′-OH in the vicinity of the bulky APPXL-I compared with other DNA polymerases under study (Figure 7). We determined hPOLη’s preference of nucleotide incorporation opposite APPXL-I to be A,G > C,T. Strikingly, incorporation opposite APPXL-Y was extremely low and could not be elucidated properly (Figure 7a). Similar results were obtained with yPOLη, which processed APPXL-Y inefficiently, while the preference of incorporation opposite APPXL-I was in the order G >> A > T >> C (Figure 7c). In the alternative substrate context, APPXL-Y was processed far more efficiently, showing the preference of incorporation in the order C >> A > G > T for both human and yeast enzymes, while the incorporation opposite to APPXL-I was again high and followed the order A,G,C > T (Figure 7b,d). Overall, the pattern of nucleotide incorporation by POLη cannot be ascribed to a single mechanism, but it is clear that the bypass of APPXLs is inaccurate and possibly involves both non-templated incorporation and template slippage, especially in the case of the bulkier APPXL-Y.

### 2.5. PrimPOL Does Not Bypass AP Site–Peptide Cross-Links but Incorporates dNMPs in a Mn^2+^-Facilitated Manner

PrimPOL, a member of the archaeal/eukaryotic primase (AEP) family, is unique among other DNA polymerases in possessing both polymerase and DNA/RNA primase activities [68,69]. Even though the latter is believed to be the primary function of this enzyme in the cell, PrimPOL is also suggested to participate in translesion synthesis. In vitro studies demonstrated that PrimPOL efficiently bypasses 8-oxoguanine, 5-formyluracil, AP site and some other lesions [68,69,70,71]. Here, we tested the ability of PrimPOL to bypass APPXLs.

**Figure 8:**
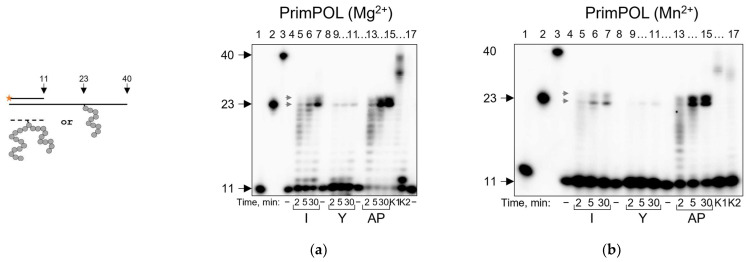

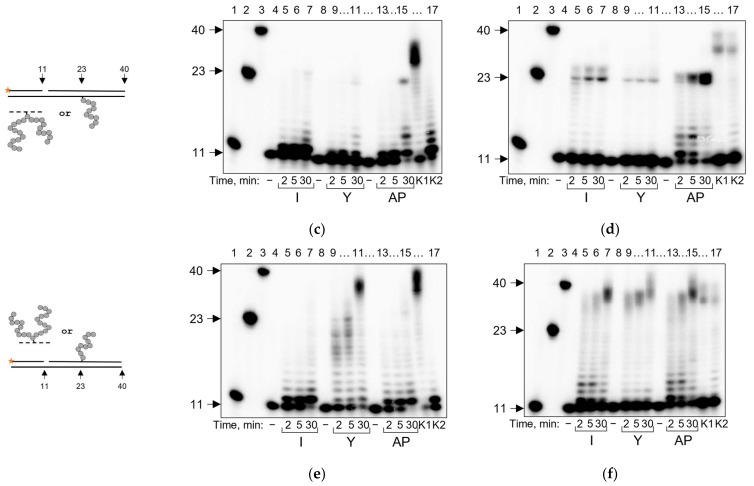
Running start synthesis by PrimPOL on APPXL-containing substrates. Schemes of the substrates are shown to the left of the gel images. Primer–template substrate, and adduct in the template: APPXL-I and APPXL-Y in the presence of Mg^2+^ (**a**) or Mn^2+^ (**b**). Primer–template–downstream strand substrate, and adduct in the template: APPXL-I and APPXL-Y in the presence of Mg^2+^ (**c**) or Mn^2+^ (**d**). Primer–template–downstream strand substrate, and adduct in the downstream strand: APPXL-I and APPXL-Y in the presence of Mg^2+^ (**e**) or Mn^2+^ (**f**). The nature of the substrate and reaction time are indicated below the gel image; I, APPXL-I; Y, APPXL-Y; AP, natural AP site. K1, undamaged primer–template substrate; K2, undamaged primer–downstream strand–template substrate; “−”, no enzyme added. In all gels: lanes 1–3, size markers with lengths corresponding to the primer (11 nt), full-size product (40 nt) and primer extended to the site of cross-linking (23 nt). Gray arrows indicate synthesis pause points.

Expectedly, PrimPOL synthesis was stalled at APPXLs, and the blocking properties of APPXLs were even stronger than those of the AP site (Figure 8a). However, PrimPOL was able to effectively reach the cross-link site (accumulation of a 23-mer product) and even incorporate one nucleotide past the adduct (24-mer product) in case of APPXL-I (Figure 8a). It has been shown that Mn^2+^ as a cofactor could increase the lesion bypass [71,72]; however, even in Mn^2+^-catalyzed reactions, PrimPOL was unable to bypass APPXLs (Figure 8b).

PrimPOL demonstrates strand displacement activity, which can be stimulated by Mn^2+^ [73]. In the presence of a downstream strand, PrimPOL was able to reach the cross-link site (Figure 8c), and Mn^2+^ facilitated nucleotide incorporation opposite the cross-link but did not lead to full lesion bypass (Figure 8d). If the cross-link was introduced into the downstream strand, PrimPOL displaced the strand containing APPXL-Y easier than the APPXL-I and AP site, which was further facilitated in the presence of Mn^2+^ (Figure 8e,f).

**Figure 9:**
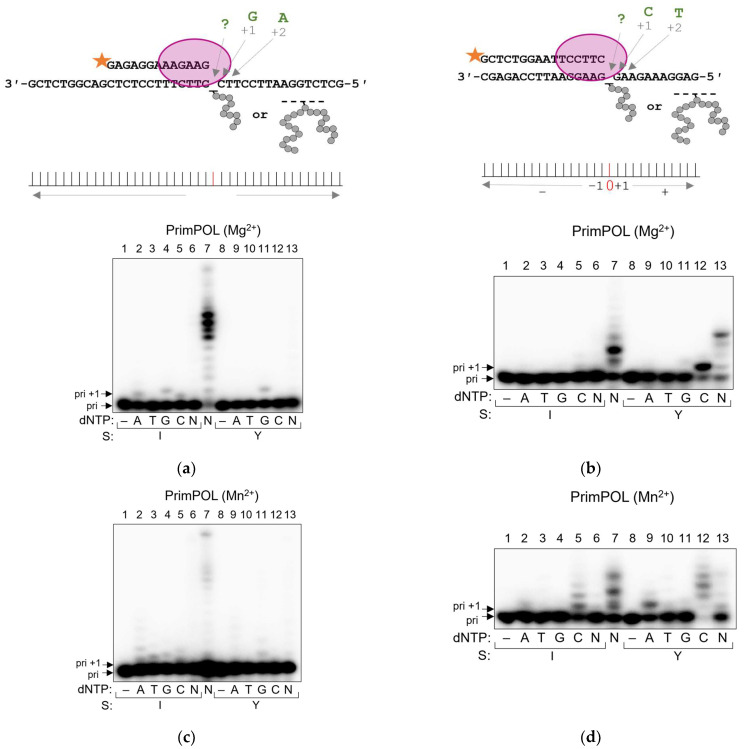
Incorporation preference of PrimPOL in standing start synthesis on APPXL-containing substrates. Schemes of the substrates are shown above the gel images. Pyrimidine-rich template (**a**) in the presence of Mg^2+^ and (**c**) in the presence of Mn^2+^. Purine-rich template (**b**) in the presence of Mg^2+^ and (**d**) in the presence of Mn^2+^. The nature of the substrate and dNTP are indicated below the gel image; I, APPXL-I; Y, APPXL-Y; N, mixture of all four dNTPs. K1, undamaged primer–template substrate; K2, undamaged primer–downstream strand–template substrate. In all gels: lane 7, undamaged substrate; “−“, no enzyme added.

Interestingly, PrimPOL’s mutagenic potential turned out to depend on the type and size of the cross-linked peptide. On the template containing APPXL-I, PrimPOL incorporated mostly A and G opposite the damage (A,G > C > T), while demonstrating a strong preference for G on the templates containing APPXL-Y (Figure 9a). Substrate switching led to a change in preference for C for both substrates; however, the incorporation of C opposite APPXL-Y was much more efficient than opposite APPXL-I (Figure 9b). Possibly, the presence of bulkier APPXL-Y facilitates lesion skipping and makes it more preferable than incorporation opposite the cross-link. Mn^2+^ generally decreased PrimPOL fidelity, which was especially noticeable in the reaction with APPXL-I, but the trend for APPXLs skipping persisted (Figure 9c,d). Interestingly, in the presence of Mn^2+^, we observed the incorporation of A along with C (Figure 9d), which possibly means that Mn^2+^ could facilitate nucleotide incorporation by PrimPOL instead of template misalignment.

## 3. Discussion

AP site–protein cross-links are an important class of DNA–protein cross-links (DPXLs) due to AP sites’ chemical properties and ubiquity. An AP site itself is one of the most mutagenic and cytotoxic forms of DNA damage as it is non-instructive and can readily be transformed to a strand break. To protect human cells from such consequences during replication, a dedicated sensor protein HMCES binds PCNA and covalently cross-links to AP sites in single-stranded DNA through a thiazolidine moiety [74,75,76,77]. This reaction is essentially irreversible, and the adduct is later resolved through proteolysis. Moreover, both natural and oxidized AP sites are intrinsically reactive and can trap a number of DNA-binding proteins [8,9,11,13,14,15,18].

As DPXLs are very bulky and chemically diverse adducts, enzymes initiating their repair do not distinguish between proteins cross-linked to DNA bases or AP sites. Therefore, DPXLs through AP sites are likely processed by SPRTN1 or related proteases to short peptides [25]. Regardless of the nature of the peptide cross-link, it is still not clear what happens next with the residual peptide attached to DNA. If not repaired in a timely manner, the peptide remnant could be encountered by a replicative, repair or translesion DNA polymerase. Despite the diversity of peptides involved, studies investigating DNA polymerase interactions with DNA–peptide cross-links generally show that some DNA polymerases can bypass DNA–peptide cross-links, with the size of the attached peptide being a decisive factor for this possibility [27,28,29,30,31,32]. However, the majority of these studies were performed for linear peptides N-terminally cross-linked to a nucleobase. The generalization of the results of such findings is complicated since different proteins can be cross-linked to DNA, meaning that the residual peptides will have different structures and shapes and will lie in different positions of the DNA helix and sequence context.

In this research, we have addressed the blocking and mutagenic properties of two peptides of different lengths and structures stably cross-linked to an AP site, APPXL-I (a 10-mer peptide conjugated through its N-terminus) and APPXL-Y (a 23-mer peptide conjugated through an internal Lys residue). In our recent study [39], we tested the ability of several non-human DNA polymerases belonging to four different structural families to carry out DNA synthesis in the presence of these adducts. Expectedly, APPXLs had strong blocking properties that were peptide-size-dependent: APPXL-Y suppressed DNA synthesis more efficiently than APPXL-I did. Interestingly, however, when compared with the natural AP site, the 10-mer I-peptide did not add significantly to its blocking properties.

Here, we have investigated the human DNA polymerases involved in DNA repair (structural Family X: POLβ and POLλ) and translesion synthesis (Family Y: POLη and POLκ, and PrimPOL from the AEP family). POLη, POLκ and PrimPOL can bypass AP sites relatively easily [45,69,71,78,79,80], whereas POLβ and POLλ are more strongly blocked but still show some residual bypass [52,81]. In many cases, this bypass involves primer–template misalignment, evident from the preference to insert a dNMP corresponding to the next nucleotide in the template. Thus, for the bulkier peptide adducts, it was interesting to assess not only the blocking properties but also the incorporation preference of the DNA polymerases.

The presence of APPXLs strongly blocked all of the polymerases to a degree at least comparable with the AP site. POLβ, POLλ and PrimPOL were unable to synthesize the full-length product on APPXL-containing templates. On the other hand, POLη, the DNA polymerase bypassing the AP site in vitro with the highest efficiency, was also able to bypass both APPXLs even if taken in lower amounts than all of the other polymerases in this study; however, compared with the natural AP site, it demonstrated the strongest drop in the amount of full-length product. POLκ, despite the relatively high concentration required, was able to bypass the AP site and APPXL-I with similar efficiencies. Generally, the bypass capability seems to correlate with the tightness of the polymerase active site. In Family X polymerases, such as POLβ and POLλ, the dRPase domain contacts DNA ahead of the moving polymerase, and restricts the volume available to accommodate a bulky adduct [82,83]. Structures of PrimPol, while having no lyase domain, also show tight contacts with the template nucleotide and the upstream template [84,85]. In contrast, Family Y DNA polymerases, including POLη and POLκ, lack the lyase domain and have a much more relaxed active site that could allow a large adducted nucleotide to rotate out and behave essentially as an AP site [86,87].

When compared with the I-peptide adduct, the bulkier Y-peptide presented a stronger block for both POLη and POLκ. Notably, the proteolysis of HMCES should give an I-type peptide, since it cross-links to AP sites through the N-terminal Cys residue, whereas non-specifically trapped proteins would statistically cross-link through internal Lys residues and produce a Y-type peptide remnant upon hydrolysis. Although the peptide size would doubtlessly matter in the bypass efficiency, an intriguing possibility is that the tendency of translesion polymerases for an easier bypass of APPXL-I adducts coevolved with the protective mechanism dependent on HMCES. The possibility of other human translesion DNA polymerases, such as POLζ, REV1 and POLι, participating in APPXL bypass remains to be explored.

If bypass occurs, the mutagenic potential of APPXLs is expected to be high, since they are non-instructive. In contrast to the Klenow fragment and phage RB69 DNA polymerase, which mostly incorporate dAMP opposite AP sites [88,89] and APPXLs [39] following the “A-rule” [90], repair and translesion DNA polymerases use different mechanisms to overcome the obstacle. Along with nucleotide incorporation opposite the damage, they use different types of template “skipping” and forming of non-canonical pairs. Generally, nucleotide insertion opposite APPXLs in standing start experiments was complicated, and more so for APPXL-Y than for APPXL-I. This was not unexpected, since a bulky adduct would obstruct polymerase binding in its vicinity. Nevertheless, we were able to establish dNMP incorporation preferences for all of the studied polymerases in two template sequence contexts (summarized in Table 1). In most cases, the results indicate a significant contribution from the template misalignment mechanism to bypass APPXLs. Interestingly, there was a trend toward easier skipping with an increasing size of the peptide, which was especially evident for PrimPOL. It should also be noted that the molecular mechanism allowing a DNA polymerase to overcome an obstacle could be different in the processive DNA synthesis mode (as in the running start experiments) when the initial primer binding is not hindered by the bulky adduct.

Unlike the adducts in the template, APPXLs in the downstream strand had little effect on the polymerases’ strand displacement activity. It was almost always comparable with, and often even superior to, the displacement of the strand containing an AP site. These observations strongly contrast the blocking ability of full-length proteins cross-linked to a downstream strand [91,92]. Presumably, cross-linked DNA-binding proteins strongly stabilize the duplex through multiple contacts with DNA, which are lost upon proteolysis. In fact, the peptide remnants may actually destabilize the duplex, which is reflected in the easier displacement of the peptide-adducted strand by the polymerases studied here.

All of the polymerases studied here operate in nuclear DNA. One interesting aspect that remains to be addressed is whether APPXLs form and interact with DNA polymerases in mitochondrial DNA. AP sites are a common type of damage in the mitochondrial genome [93,94], and recently have been shown to trap mitochondrial transcription factor A (TFAM), an abundant histone-like protein [95]. However, it is not known whether the proteolysis-dependent repair operates in mitochondria, and homologous recombination was suggested to be the main route of mitochondrial DPXL repair [96]. Of all the specialized translesion polymerases, only PrimPol has so far been shown to operate in mitochondria [69,97], while the replication and repair are dependent on DNA polymerase γ. Thus, mitochondrial DNA tolerance to cross-linked proteins could depend on PrimPol, which can bypass DPXLs or re-prime DNA synthesis downstream of them [72].

The cellular consequences of APPXLs ultimately depend on the balance between their repair (possibly by APEX1 AP endonuclease [39]) and translesion synthesis. Apart from well-documented APEX1 participation in BER complexes, which may be transient or stable and involve POLβ [98,99,100], APEX1 interacts with PCNA [101,102], making it possible that active replication complexes stalled on DPXLs or APPXLs already carry the repair protein or attract it to the damage site. Moreover, a proteomic screen of APEX1 interactors revealed translesion polymerases POLκ and POLι, as well as RAD9B, an alternative subunit of the 9-1-1 clamp complex replacing PCNA at damaged sites to facilitate DNA repair and translesion synthesis [103]. However, the kinetics of APPXL removal by APEX1 is slow [39], and so it remains to be seen whether the presence of competing repair activity is sufficient to prevent pro-mutagenic translesion synthesis over these sites.

To summarize, AP site–peptide cross-links are a common but underexplored type of DNA damage. They are non-instructive and strongly blocking, and thus are expected to be highly cytotoxic and mutagenic. We have shown that, in comparison with regular AP sites, some APPXLs would present a problem even for dedicated translesion DNA polymerases, although POLη and POLκ may bypass them with limited efficiency. These observations warrant further investigations of mechanisms of APPXL repair and their bypass by other polymerases.

## 4. Materials and Methods

### 4.1. Oligonucleotides and Enzymes

*E. coli* Fpg protein [40], human OGG1 protein [104], human POLκ (catalytic core, residues 1–560) [105], POLβ [106], POLλ [107], PrimPol [72], human DNA polymerase η (hPOLη) [72] and yeast DNA polymerase η (yPOLη) [108] were essentially overexpressed and purified as described. *E. coli* uracil–DNA glycosylase (Ung) was purchased from SibEnzyme (Novosibirsk, Russia) and trypsin was purchased from Samson-Med (St. Petersburg, Russia). Oligonucleotides (Table 2) were synthesized in-house from commercially available phosphoramidites (Glen Research, Sterling, VA, USA).

### 4.2. Model AP Site–Peptide Conjugates’ Preparation

Stable DNA–protein conjugates with AP site with Fpg or with OGG1 proteins were obtained as described by cross-linking the enzyme to the 40oG//28down or 28oG//21comp duplexes [39,72,91,92]. The difference in the length of the strands in the starting duplexes permitted easy electrophoretic separation of the modified, non-modified and complementary strands. Then, the cross-links were subjected to trypsinolysis (2 μg of trypsin per 3–6 nmol of starting protein, 4 h at 37 °C), purified via denaturing polyacrylamide electrophoresis, desalted via reverse-phase chromatography (C18 Isolute sorbent, Biotage, Uppsala, Sweden) and characterized via MALDI mass spectrometry, as described in [39].

### 4.3. Running Start Assay

APPXL-containing substrates were assembled by annealing the 40-mer template strand with the 11-mer [^32^P]-labeled primer (run_pri) and, if necessary, with the downstream strand (28down), as described in [91,92]. The cross-link was placed in either the template or the downstream strand. To prepare the AP-site-containing substrates, oligonucleotides were assembled in the same way, but with a uracil-containing 40-mer template strand (40U), and treated with Ung for 20 min at 37 °C immediately before the reaction. The reaction mixture (40 μL) contained 50 nM DNA substrate, dNTPs (each at 250 μM) and DNA polymerase in the appropriate reaction buffer (Table 3). The reaction was allowed to procced at 37 °C; aliquots were withdrawn at 2, 5 and 30 min, mixed with an equal volume of the stop solution (80% formamide, 20 mM Na-EDTA, 0.1% xylene cyanol, 0.1% bromophenol blue) and heated for 2 min at 95 °C. The reaction products were separated via electrophoresis in 20% denaturing polyacrylamide gel and visualized using a Typhoon FLA 9500 phosphorimager (GE Healthcare, Chicago, IL, USA). Quantity One v4.6.8 (Bio-Rad Laboratories, Hercules, CA, USA) was used to quantify the band intensity.

### 4.4. Standing Start Assay

APPXL-containing substrates were assembled by annealing the 40-mer lesion-bearing template strand with the 13-mer [^32^P]-labeled primer (stand_pri_1). To provide an alternative sequence context, the 28-mer lesion-bearing template strand (derived from the 28oG oligonucleotide) was annealed to the 16-mer [^32^P]-labeled primer (stand_pri_2). The reaction mixture contained 20 nM DNA substrate, 200 μM dNTPs (A, C, G, T or each) and DNA polymerase in the appropriate reaction buffer (Table 3). The reaction was allowed to procced at 37 °C for 30 min, and was then terminated and analyzed as described above.

## Figures and Tables

**Table 1:** Overview of DNA polymerases’ preference for nucleotide incorporation opposite APPXLs and suggested mechanisms of damage bypass.

Polymerase	Pyrimidine-Rich Template 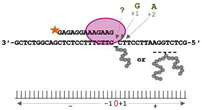	Purine-Rich Template 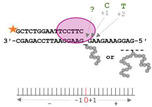	Suggested Mechanism
POLβ	G > A > C > T	C > A > T > G	+1 dNTP-stabilized misalignment > “A rule”.
POLλ	G >> A > C > T	C >> T > A > G	+1 dNTP-stabilized misalignment >> +2 dNTP-stabilized misalignment.
POLκ	A > G,C > T	C > A,T > G	+1 dNTP-stabilized misalignment >> +2 dNTP-stabilized misalignment, “A rule”.
hPOLη	A,G > T,C (APPXL-I)	A,G,C > T (APPXL-I)	Inaccurate. Likely several mechanisms.
	G > T,C,A (APPXL-Y)	C >> A > G > T (APPXL-Y)	
yPOLη	G >> A > T >> C (APPXL-I)	A,G,C > T (APPXL-I)	
	G > T,C,A (APPXL-Y)	C >> A > G > T (APPXL-Y)	
PrimPOL	A ~ G > C > T (APPXL-I/Mg^2+^)	C > A,G,T (APPXL-I/Mg^2+^)	Size dependent: “A rule” for APPXL-I, +1 dNTP-stabilized misalignment for APPXL-Y.
	G >> A,T,C (APPXL-Y/Mg^2+^)	C >> A,G,T (APPXL-Y/Mg^2+^)	
	A ~ G ~ C ~ T (APPXL-I/Mn^2+^)	C >> A > T,G (APPXL-I/Mn^2+^)	
	G > A,C > T (APPXL-Y/Mn^2+^)	C >> A > T,G (APPXL-Y/Mn^2+^)	

**Table 2:** Oligonucleotides used in this work.

ID	Sequence, 5′→3′	Length	Modification (X)
40oG	GCTCTGGAATTCCTTCXCTTCTTTCCTCTCGACGGTCTCG	40	8-oxoguanine
40U	GCTCTGGAATTCCTTCXCTTCTTTCCTCTCGACGGTCTCG	40	uracil
28down	GAGGAAAGAAGCGAAGGAATTCCAGAGC	28	
28oG	GAGGAAAGAAGXGAAGGAATTCCAGAGC	28	8-oxoguanine
40temp	GCTCTGGAATTCCTTCCCTTCTTTCCTCTCGACGGTCTCG	40	
40comp; 40marker	CGAGACCGTCGAGAGGAAAGAAGCGAAGGAATTCCAGAGC	40	
21comp	GGAATTCCTTCCCTTCTTTCC	21	
23marker	CGAGACCGTCGAGAGGAAAGAAG	23	
run_pri; 11marker	CGAGACCGTCG	11	
stand_pri_1	GAGAGGAAAGAAG	13	
stand_pri_2	GCTCTGGAATTCCTTC	16	

**Table 3:** Assay buffers and reaction conditions.

Enzyme	Concentration in Running Start Assay, nM	Concentration in Standing Start Assay, nM	Reaction Buffer	Reference
POLβ	200	300	20 mM Tris–HCl (pH 7.6), 10 mM MgCl_2_, 1 mM DTT	[46]
POLλ	200	200	50 mM Tris–HCl (pH 8.5), 2.5 mM MgCl_2_, 2% glycerol, 1 mM DTT, 100 µg/mL BSA	[46], modified after [109]
POLκ	100	100	50 mM Tris–HCl (pH 7.5), 5 mM MgCl_2_, 1 mM DTT	[46]
hPOLη, yPOLη	50	120	30 mM HEPES–NaOH (pH 7.4), 50 mM NaCl, 1 mM MgCl_2_, 1 mM DTT, 100 µg/mL BSA	[92]
PrimPOL	200	500	30 mM HEPES (pH 7.2), 10 mM MgCl_2_ or 0.5 mM MnCl_2_, 5% glycerol, 100 µg/mL BSA	[72]

## Data Availability

The data presented in this study are available in the article.
